# Intelligent Identification for Rock-Mineral Microscopic Images Using Ensemble Machine Learning Algorithms

**DOI:** 10.3390/s19183914

**Published:** 2019-09-11

**Authors:** Ye Zhang, Mingchao Li, Shuai Han, Qiubing Ren, Jonathan Shi

**Affiliations:** 1 State Key Laboratory of Hydraulic Engineering Simulation and Safety, Tianjin University, Tianjin 300072, China; jgzhangye@tju.edu.cn (Y.Z.); hs2015205039@tju.edu.cn (S.H.); qbren@tju.edu.cn (Q.R.); 2 College of Engineering, Louisiana State University, Baton Rouge, LA 70803, USA; jshi@lsu.edu

**Keywords:** rock-mineral microscopic images, deep learning, model stacking, transfer learning, CNN, machine learning

## Abstract

It is significant to identify rock-mineral microscopic images in geological engineering. The task of microscopic mineral image identification, which is often conducted in the lab, is tedious and time-consuming. Deep learning and convolutional neural networks (CNNs) provide a method to analyze mineral microscopic images efficiently and smartly. In this research, the transfer learning model of mineral microscopic images is established based on Inception-v3 architecture. The four mineral image features, including K-feldspar (Kf), perthite (Pe), plagioclase (Pl), and quartz (Qz or Q), are extracted using Inception-v3. Based on the features, the machine learning methods, logistic regression (LR), support vector machine (SVM), random forest (RF), k-nearest neighbors (KNN), multilayer perceptron (MLP), and gaussian naive Bayes (GNB), are adopted to establish the identification models. The results are evaluated using 10-fold cross-validation. LR, SVM, and MLP have a significant performance among all the models, with accuracy of about 90.0%. The evaluation result shows LR, SVM, and MLP are the outstanding single models in high-dimensional feature analysis. The three models are also selected as the base models in model stacking. The LR model is also set as the meta classifier in the final prediction. The stacking model can achieve 90.9% accuracy, which is higher than all the single models. The result also shows that model stacking effectively improves model performance.

## 1. Introduction

At present, image pattern recognition is widely used in image data analysis, especially in earth sciences. Analysis of microscopic images of rock, and mineral classification and identification are fundamental tasks in geological research. The first step of studying a rock-mineral sample in the lab is also a significant way to determine the rock-mineral type and properties. It is also the basis of the geochemical analysis, such as in major, minor, and isotope element tests. To date, rock-mineral microscopic image identification has been conducted manually by engineers and scholars, which depends on the operators’ experience and skill. It is inefficient and time-consuming. Moreover, the result is determined largely by the conductor’s knowledge. Incorrect rock-mineral recognition impairs subsequent work, which may lead to wasted resources and economic loss. It is crucial to develop efficient, robust, and objective automatic recognition techniques for rock-mineral microscopic images.

Researchers have combined computer vision and machine learning to analyze the automatic classification and identification of rock-mineral microscopic images. Singh et al. [1] extracted 27 features from a thin section of a rock sample and applied a multilayer perceptron neural network to predict the test data, which achieved 92.2% accuracy. Młynarczuk et al. [2] and Ślipek and Młynarczuk [3] applied four algorithms—nearest neighbor, K-nearest neighbor, nearest mode, and optimal spherical neighborhoods—to classify rock-mineral microscopic images. Ładniak and Młynarczuk [4] adopted a clustering algorithm to classify thin rock-mineral sections, which achieved about 100% accuracy. Aligholi et al. [5] extracted seven optical features to classify minerals. Mollajan et al. [6] integrated a fuzzy fusion of support vector machine (SVM), K-nearest neighbors (KNN), and radial basis function (RBF) to identify pore type. SVM was outstanding in these models, with an accuracy of 94.4%. Chauhan et al. [7] compared seven machine learning methods, including unsupervised, supervised, and ensemble clustering techniques, to process X-ray microtomographic rock images. Shu et al. [8] applied unsupervised learning methods to analyze manual features for rock image classification. Aligholi et al. [9] proposed a mineral classification scheme using color tracking. Compared to conventional classification methods, the color-based procedure can provide reliable identification results. Galdames et al. [10] used the SVM and voting process based on 3D laser range features to classify rock images. Compared to manual identification for a rock-mineral thin section, machine learning methods have advantages. Researchers focused on rock-mineral color and texture, and took related features as inputs for machine learning. However, the inputs for machine learning need to be preprocessed first, which is called feature engineering. Feature engineering has a significant influence on the results of machine learning methods; even if the same machine learning model is adopted, the result will probably not be identical with different types of feature engineering.

Deep learning [11,12,13,14,15,16] has been increasingly prevalent in image analysis due to the ability of automatic feature extraction. It has been applied in many fields [17,18,19]. Moreover, deep learning is also employed in rock-mineral microscopic image identification. Jiang et al. [20] applied the convolutional neural network (CNN) to extract sandstone image features. Segmentation based on deep learning was also adopted in rock-mineral type determination [21,22]. However, there are challenges and limitations in deep learning. Deep learning is data-hungry and its architecture is complex. Even though in the same class, thousands of labeled images are required in deep learning model training. It is hard to collect and label such a large quantity of data, which makes it infeasible for rock-mineral microscopic image identification. Moreover, training a large deep learning model from scratch has a significant cost in terms of computation resources.

Transfer learning provides a new approach to deep learning model application for rock-mineral images. The transfer learning model can be established based on the similarity between different tasks. The obtained knowledge can be transferred to a related domain with little change using transfer learning. In the application of deep learning models using transfer learning, the weights of the models can be reused in new model construction [23]. There are two advantages of transfer learning using a pre-trained deep learning model. First, the outstanding-performance model can be trained only using a small dataset. Second, model training is fast using transfer learning and the pre-trained model. Although there are insufficient images in each class, a good-performance model can be obtained using transfer learning [24,25,26]. In geological image analysis, transfer learning and deep learning models have been adopted. Li et al. [27] classified sandstone microscopic images automatically using transfer learning. Zhang et al. [28] applied Inception-v3 to classify different geological structures, which proved the effectiveness of the deep learning model. The research indicates the effectiveness of deep learning in feature extraction. Transfer learning has been proved to be an efficient analysis method.

Transparent mineral is the main constituent in rock. Accurate rock-mineral identification makes it possible to conduct microscopic quantitative analysis, which is the basis of rock-mineral research. If the trained model can achieve human-level accuracy on similar minerals, it can be applied in rock-mineral microscopic image analysis with higher efficiency than humans. In this research, the orthogonal polarization spectral (OPS) images of K-feldspar (Kf), perthite (Pe), plagioclase (Pl), and quartz (Qz or Q) are selected as the study sample. The four minerals are the common rock-forming minerals, which are widely distributed in metamorphic rocks. However, there are some similarities in their OPS image features. We integrated the deep learning model and machine learning methods to undertake a comprehensive analysis of the rock-mineral OPS images. The results indicate the Inception-v3 model can extract effective features. The machine learning algorithms based on perceptron are proved to be outstanding. Model stacking is also proposed to improve model performance further.

## 2. Methodology

**Figure 1:**
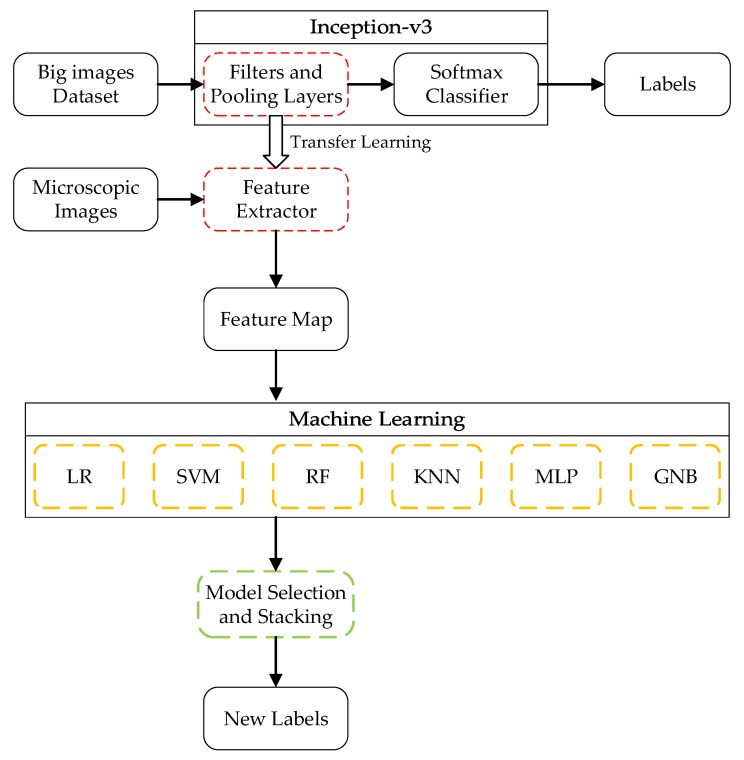
Overall framework for rock-mineral microscopic images identification.

In this research, transfer learning using a deep learning model and several machine learning algorithms is applied to identify rock-mineral microscopic images. The deep learning model is set as the pre-trained model, which is trained using a convolutional neural network (CNN). The features are generated by transfer learning using the Inception-v3 model. Based on the extracted features, logistic regression (LR), support vector machine (SVM), random forest (RF), k-nearest neighbors (KNN), multilayer perceptron (MLP), and gaussian naive Bayes (GNB) are applied to establish the models. Through the comparison of the different models’ performance, the outstanding models can be selected as the base models in model stacking. The schematic for rock-mineral microscopic image identification is shown in Figure 1.

### 2.1. Convolutional Neural Network

The deep learning model is trained on some data set using convolutional neural network architecture. A convolutional neural network (CNN) is a kind of feed-forward neural network, which is designed for unstructured data identification, such as image, text, and sound. It usually consists of convolutional layers and pooling layers. Compared to the fully connected layer, each neuron in the convolutional layer is connected to certain neurons in the previous layer, which is a small and squared area in the image pixel matrix. The size of the small area in the matrix is called the receptive field, which spans the dimensions of height and width in the image. There are no special parameters for image depth. Color information is also significant in model training. As a consequence, the convolutional layer should be conducted across the whole color space.

The neurons in one convolutional layer share the same weight to recognize certain patterns from the previous layer. The certain patterns should have translation invariance, which means the features should be independent of their coordinates in the image. As a result, all the neurons in the same kernel should share the same parameters, which is called parameter sharing. Since each kernel can just recognize a certain pattern, there are several kernels in one layer to identify multiple patterns in different places of the image. A pooling layer is also a significant concept in CNN, and can decrease the feature dimension and the computation cost. They are also connected to a special region of the previous layer, like the convolutional layer. Compared to the convolutional layers, pooling layers are determined by their own set rather than the parameters in the model training process. In CNN, max and mean pooling are commonly used. The computation is processed in each neuron in the CNN as follows:

(1) $$f\left(x\right)=act\left(\sum_{i,j}^{n}\theta_{\left(n-i\right)\left(n-j\right)}x_{ij}+b\right),$$

where f(x) is the output, act is the activation function, θ is the weight matrix, $x_{ij}$ is the input, and b is the bias.

### 2.2. Inception-v3 and Transfer Learning

**Figure 2:**
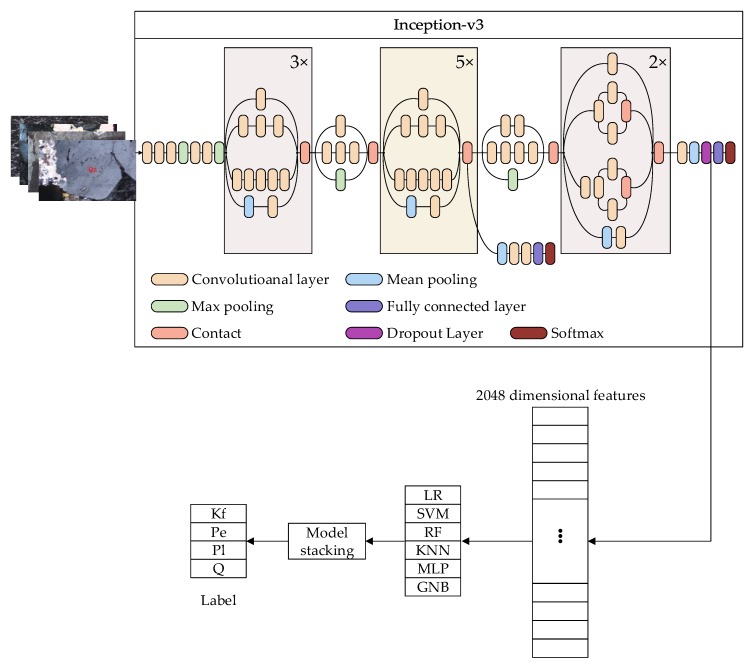
Transfer learning based on Inception-v3.

Compared to the GoogLeNet (Inception-v1), Inception-v3 [17] has made large progress. It integrates all the updates in Inception-v2. Furthermore, there are some new improvements in Inception-v3. In model design, the optimization-SGD (stochastic gradient descent) is replaced by RMSProp (root mean square prop). In the classifier, the LSR (label-smoothing regularization) is added after the fully connected layer. In the convolutional layer, the 7 × 7 kernel is replaced by a 3 × 3 kernel. Normalization is also used and regularization is added to the loss function to avoid overfitting. Figure 2 shows the compressed view of the Inception-v3 model. At the beginning of the model, 3 convolutional layers and 2 pooling layers are set, then 2 convolutional layers and 1 pooling layer are set, and, finally, it follows 11 mixed layers, the dropout layer, the fully connected layer, and the softmax layer.

The operations of convolution and padding are conducted repeatedly on the image in each layer. Table 1 shows the specific process of convolution and padding. The data transformation is presented. Before the softmax layer, the image is converted to a 2048-dimension vector, as shown in Figure 1. The high-dimension vector includes several geometric and optical features. Some of the features will be presented in Section 4. 

In most cases of machine learning application, the model is established from scratch even though there is an existing model based on similar data. The repeated construction of the model is a waste of resources. Considering the similarity of the different models, transfer learning can be applied to establish a new model using the obtained knowledge. Meanwhile, there have been two problems in deep learning model training: the data is often insufficient and the computation is slow. However, a good-performance model can be trained using little data and a pre-trained model. Considering the relationship between the source domain and the target domain, the new model can be established using transfer learning.

In the process of deep learning model retraining, the extracted features are adopted to train the new model [29]. As shown in Figure 2, the images are the input; all the convolutional and pooling layers are reused in the new model training. In other words, Inception-v3 is taken as a feature extractor, while the extracted 2048-dimension features are fed to multi-machine learning algorithms rather than the softmax layer. The new model will be established using model stacking. The whole process is shown in Figure 2. In our research, K-feldspar (Kf), perthite (Pe), plagioclase (Pl), and quartz (Qz or Q) images are employed.

### 2.3. Machine Learning Algorithms

Recently, machine learning has been increasingly used in classification and pattern recognition. In our research, the input data are complex non-linear features, which are generated using the Inception-v3 model. LR, SVM, RF, KNN, MLP, and GNB are selected to establish the identification model. LR, SVM, and MLP are based on perceptron; RF is a tree model; KNN is a non-parameter model; and GNB is a model based on probability. It is beneficial for the optimized model search to high-level features (deep model features) training in different machine learning methods.

#### 2.3.1. Logistic Regression (LR)

Logistic Regression is a generalized linear model. Some variables in LR are obtained by linear models. The nonlinear sigmoid function is used to map predictions to probabilities. The LR model can be expressed as Equation (2):

(2) $$h_{\theta }\left(x\right)=\frac{1}{1+e^{-\theta^{T}x}},$$

where $h_{\theta }\left(x\right)$ is the probability. The probability values range from 0 to 1, which is an S-shaped curve and splits the space into two equal parts. $\theta^{T}x$ is the linear combination of several related variables.

#### 2.3.2. Support Vector Machine (SVM)

The support vector machine (SVM) was proposed by Cortes and Vapnik [30]. Suppose that the data is (*x*_1_, *y*_1_) (*x*_2_, *y*_2_) … (*x_n_*, *y_n_*); *x* is the input vectors; *n* is the number of the training samples; and *y* is the label, where *y* = {−1, +1}. In the classification problem using SVM, the target is to search the hyperplane to maximize the margin between the two support vectors. The objective function and the constraint are shown in Equation (3):

(3) $$\left\{ \begin{array}{l} min\;\frac{1}{2}{\left\|w\right\|}^{2}+C\sum_{i=1}^{n}\xi_{i}\\ s.t.\;y_{i}\left[\left(wx_{i}\right)+b\right]\geq 1-\xi_{i}\;\;\left(i=1,2,\ldots ,n\right) \end{array}\right.,$$

where *w* is the adjustable weight, ‖w‖ is the Euclidean norm of the vector, $\xi_{i}$ is the slack variable, which is used to relax the constraints, and *C* is the penalty parameter, which makes a trade-off between margin and misclassification.

#### 2.3.3. Random Forest (RF)

Random forest (RF) was proposed by Breiman [31], and combines multiple decision trees. Compared to traditional bagging, the base learning model in RF is the decision tree, while the training process is determined by random attributes selection. The x-dimension vector is fed to the RF model; then the K decision trees ${\left\{T\left(x\right)\right\}}_{1}^{K}$ generate and are independent of each other. The RF model is expressed in Equation (4). Each tree will make a prediction and voting is applied to make a decision. The label predicted by the majority of the decision trees is regarded as the final prediction.

(4) $${\hat{f}}_{\gamma f}^{K}\left(x\right)=\frac{1}{K}\sum_{K=1}^{K}T\left(x\right).$$

To reduce the correlation of the different decision trees, bootstrap aggregating is adopted. The decision trees in a different training subset are generated, which can improve the generalization and robustness of the model.

#### 2.3.4. K-Nearest Neighbors (KNN)

K-nearest neighbors (KNN) is a non-parametric and simple algorithm. It is a lazy algorithm that does not generalize the training data. The steps in KNN can be described as the following:

- Calculate the distance between the training and the test data;
- Arrange the distance from smallest to largest;
- Select K minimum-distance points;
- Calculate the frequency of the K points in each group;
- Return the label with the highest frequency of K and the label is the prediction.

If the training dataset has *n* attributes, the distances of two datasets can be calculated based on these attributes. The Euclidean distance is usually selected. For example, two datasets are given as *X* = (*x*_1_, *x*_2_, …, *x_n_*) and *Y* = (*y*_1_, *y*_2_, …, *y_n_*). The Euclidean distance between *X* and *Y* is shown in Equation (5):

(5) $$d\left(X,Y\right)=\sqrt{\sum_{i=1}^{n}{\left(x_{i}-y_{i}\right)}^{2}}.$$

#### 2.3.5. Multilayer Perceptron (MLP)

**Figure 3:**
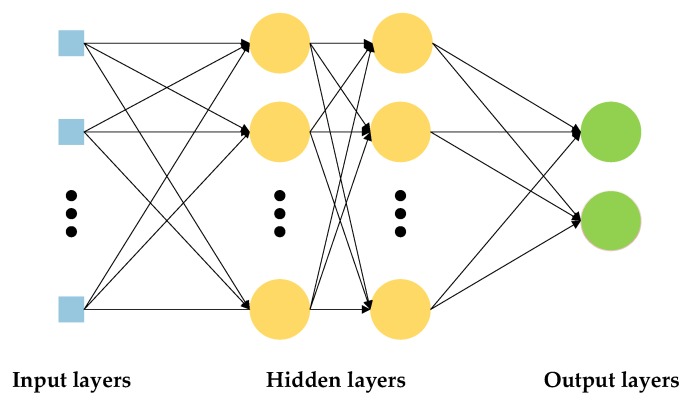
MLP model structure.

Multilayer perceptron (MLP) is a computing network that is inspired by biological neural networks. Generally, the structure of MLP consists of three significant layers, which are the input layer, the hidden layer, and the output layer, as shown in Figure 3. The number of neurons in the input, hidden and output layers, network architecture, and the learning rate are the parameters to be selected to develop an MLP model. The MLP model is trained with a set of known input data and output data. The training process continues until the network output matches the desired output. Changing the weights and biases shall reduce the error between the network output and the target output. The training process is terminated automatically when the error falls below a threshold or the maximum epochs are exceeded.

#### 2.3.6. Gaussian Naive Bayes (GNB)

Gaussian naive Bayes (GNB) is a supervised learning method, which is based on Bayes’ theory. GNB supposes that the features are independent of each other. For the label *y* and the features *x*_1_ to *x_n_*, the probability relationship can be expressed as follows:

(6) $$P\left(y|x_{1},\ldots x_{n}\right)=\frac{P\left(y\right)P\left(x_{1},\ldots x_{n}|y\right)}{P\left(x_{1},\ldots x_{n}\right)}.$$

In Bayes’ theory, the features are independent of each other. Equation (6) can be expressed as Equation (7):

(7) $$P\left(y|x_{1},\ldots x_{n}\right)=\frac{P\left(y\right)\prod_{i=1}^{n}P\left(x_{i}|y\right)}{P\left(x_{1},\ldots x_{n}\right)},$$

$P(x_{1},\ldots x_{n})$ is a constant, thus we can analyze Equation (8).

(8) $$\begin{array}{l} P\left(y|x_{1},\ldots x_{n}\right)\propto P\left(y\right)\prod_{i=1}^{n}P\left(x_{i}|y\right)\\ \;\;\;\;\;\;\;\;\;\;\;\;\;\;\;\;\;\;\;\;\;\;\;\;\;\;\;\;\;⇓\\ \hat{y}=\underset{y}{argmax}P\left(y\right)\prod_{i=1}^{n}P\left(x_{i}|y\right) \end{array},$$

In GNB, the features obey the Gauss distribution, as shown in Equation (9):

(9) $$P\left(x_{i}|y\right)=\frac{1}{\sqrt{2\pi \sigma_{y}^{2}}}\exp\left(-\frac{{\left(x_{i}-u_{y}\right)}^{2}}{2\sigma_{y}^{2}}\right).$$

### 2.4. K-Fold Cross-Validation

**Figure 4:**
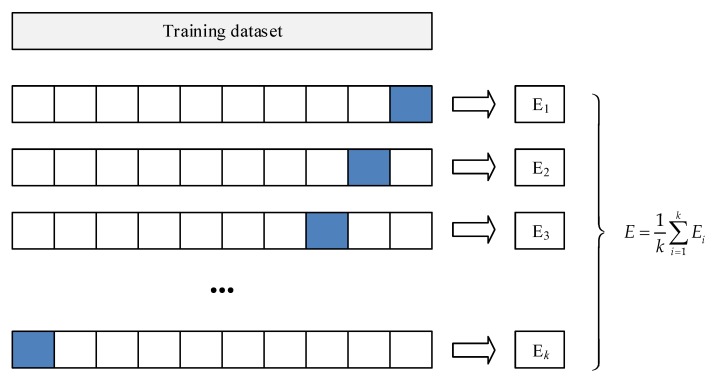
*k*-fold cross-validation.

In *k*-fold cross-validation, the original data is randomly divided into *k* equal-sized subsamples. In the *k* subsamples, one subsample is set as the validation data, and the remaining *k* − 1 subsamples are taken as the training data. The cross-validation process is repeated *k* times, namely, *k* folds. Each of the *k* subsamples is taken as the validation data for just one time. The results from all folds can make a comprehensive evaluation. The advantage of this method is that all data can be both training and validation data, and each one is used for validation exactly once. The *k*-fold cross-validation is more objective than the simple cross-validation. The process of the *k*-fold is shown in Figure 4. The blue fold is set as validation. The mean value, E, of the *k*-fold is taken as the final evaluation.

### 2.5. Model Stacking

Model stacking [32] is one of the model ensemble methods, which is not the same as bagging or boosting. Two-stage training is conducted to establish the model. The process of model stacking is as following:

- The base models are trained on the same dataset using *k*-fold cross-validation (usually *k* = 5 or 10);
- The *m* base models with significant performance are selected to make a prediction and the *k*-fold cross-validation is also employed;
- The mean value of the base model’s *k*-fold cross-validation predictions are taken as the new features;
- Based on the new features, a senior model can be trained.

**Figure 5:**
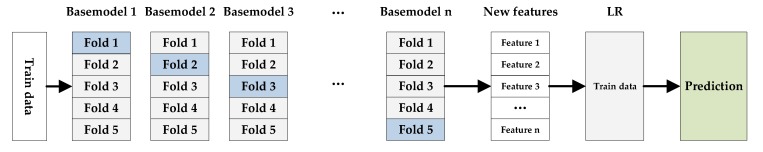
Model stacking.

It is obvious that there are two stages in model stacking. In the first stage, *m* base classification models are selected to build new features. The robustness of the new features as training data is guaranteed by the adoption of 5-fold cross-validation. In the second stage, LR is commonly chosen as the meta-model to build the model and make a final prediction. The process of model stacking is shown in Figure 5.

## 3. Data Collection and Preprocessing

**Figure 6:**
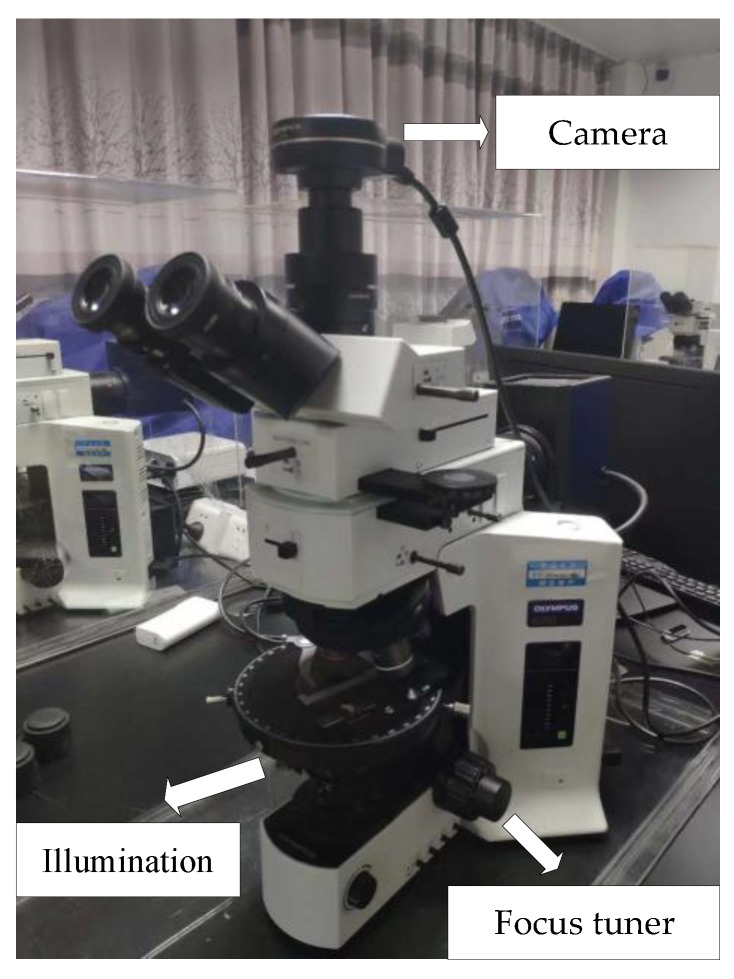
Microscope.

In the field, a rock commonly consists of an aggregate of two or more different minerals. In the identification of the rock-mineral thin section, the main task is to distinguish each mineral and recognize them under the microscope. In this research, 1-mm thin-section images of K-feldspar (Kf), perthite (Pe), plagioclase (Pl), and quartz (Qz or Q) are applied to establish the model; the microscope is shown in Figure 6. The mineral images can be obtained using the camera on the top of the microscope. Under the microscope carrier, there is a halogen lamp applied as the light source. The focus can be tuned using the knob beside the microscope.

**Figure 7:**
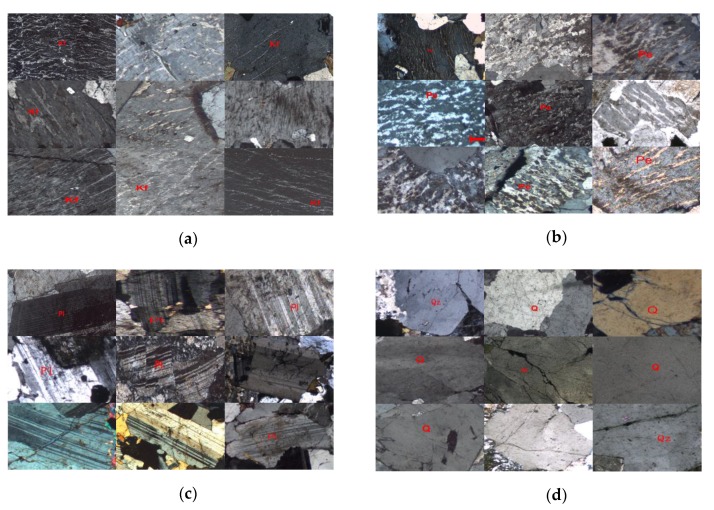
The four cut mineral microscopic images: (**a**) Kf; (**b**) Pe; (**c**) Pl; (**d**) Qz.

The four minerals exist together with other minerals. The target images will be cut from the whole thin section images. The target mineral image should cover most of the region in the cut image. Finally, there are a total of 481 images in all the classes. The specific information is listed in Table 2. In 10-fold cross-validation, the data is divided into training and validation datasets with a 90/10 split in each cycle. The four minerals’ thin section images are shown in Figure 7. Nine samples in each group are presented.

## 4. Model Establishment and Evaluation

**Figure 8:**
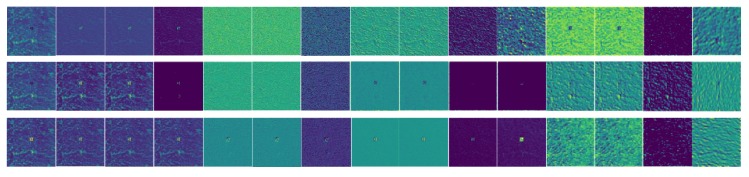
The feature map visualization of Kf.

In the process of OPS microscopic rock-mineral image feature extraction using Inception-v3, there is no special limitation to the raw data. The size of the images can be processed to be 299 × 299 × 3 automatically before training, where 299 denotes the height and width of the image size, and 3 denotes the three channels of RGB (red, green, and blue). The feature map visualization based on the image in Figure 7a is shown in Figure 8. It shows 3 feature maps of each layer in the first 15 layers. The process of feature extraction is presented. In the different layers, the extracted features are different. It is easy to see that some features, such as chromatic aberration and texture, can be extracted using the Inception-v3 model.

Based on the extracted features, LR, SVM, RF, KNN, MLP, and GNB are adopted to establish the prediction model using Scikit-learn [33]. The data is split into training data and test data with a 90/10 ratio in every fold of cross-validation. Most of the parameters of the algorithm are default ones. The selected parameters of each machine learning method are listed in Table 3.

Based on the model training parameters set in Table 3, all the models are evaluated using 10-fold cross-validation. Because of the 10-fold cross-validation application, the mean value and standard deviation of the accuracy can be used to present the model performance. The accuracy and the accuracy standard deviation of each model are summarized in Table 4. It can be found that LR, SVM, and MLP have a significant effect on extracted features, with higher accuracy than the other models. The accuracy is about 90.0%.

Since LR, SVM, and MLP are outstanding among all the models, the three models are employed as the base models in model stacking. The parameters of the three models in Table 3 are also employed in model stacking. In the first training stage, the three models generate new features, and 5-fold cross-validation is applied to show objective accuracy; in the second training stage, the final model is established using LR. The model selection is significant to the performance of the stacking model. All the base models should have outstanding performance. The base model with low accuracy has a negative influence on the final result. There are no special constraints to the new features after model selection. The evaluation results of the stacking model and the three single models are shown in Table 5.

Table 5 shows that the stacking model has the highest accuracy and the accuracy standard deviation is relatively low. In the process of model stacking, the parameters of all the involved models are fixed. It proves that model stacking can improve the prediction performance without parameter tuning again. Meanwhile, considering the accuracy standard deviation, the stacking model is relatively stable.

## 5. Conclusions

In this research, the deep learning model Inception-v3 is adopted to extract high-level features of quartz and feldspar microscopic images. Different machine learning methods and 10-fold cross-validation are adopted. The highest accuracy of the single model, SVM, is 90.6% and the lowest accuracy is that of GNB, of about 78.0%, which indicates that the features extracted by Inception-v3 are effective. The deep learning models and the extracted features can be applied in smart identification of rock-mineral thin sections.

Furthermore, based on the extracted features, the six machine learning methods—LR, SVM, MLP, RF, KNN, and GNB—are applied to make a prediction. The result shows LR, SVM and MLP have a significant performance, which means the methods based on the perceptron are effective on the high-level features. The accuracy of the three models is about 90.0%.

Since LR, SVM, and MLP have outstanding performance, the three models are selected as the base models for model stacking. The 5-fold cross-validation is also applied to evaluate the stacking model. The result shows that the stacking model has a better performance than the single models, with an accuracy of 90.9%. It proves that model stacking is also effective for high-dimensional features.

In the future, more types of mineral samples should be added to train the model. Then, the microscope, the computer, and the model can be integrated to identify rock-mineral thin sections automatically.

## Figures and Tables

**Table 1:** The outline of the Inception-v3 model.

Type	Patch Size/Stride or Remarks	Input Size
Conv	3 × 3/2	299 × 299 × 3
Conv	3 × 3/1	149 × 149 × 3
Conv Padded	3 × 3/1	147 × 147 × 32
Pool	3 × 3/2	147 × 147 × 64
Conv	3 × 3/1	73 × 73 × 64
Conv	3 × 3/2	71 × 71 × 80
Conv	3 × 3/1	35 × 35 × 192
3 × Inception	1 × 1 and 3 × 3/1	35 × 35 × 288
5 × Inception	*n* × 1, 1 × *n*, and *n* × *n*/2	17 × 17 × 768
2 × Inception	1 × 1, 1 × 3, 3 × 1, and 3 × 3/2	8 × 8 × 1280
Pool	8 × 8	8 × 8 × 2048
Linear	Logits	1 × 1 × 2048
Softmax	Classifier	1 × 1 × 1000

**Table 2:** The numbers of sample data of rock-mineral microscopic images.

Mineral	Number of Images in Each Class	Total Number of Images
Kf	116	-
Pe	122	481
Pl	122	-
Qz	121	-

**Table 3:** The parameters set in the models.

Method	Parameters	Value
LR	Solver	Newton-cg
	Multi_class	Multinomial
LinearSVC	C	1.0
RF	N_jobs	4
	Criterion	Entropy
	N_estimators	70
	Min_samples_split	5
KNN	N_neighbors	1
	N_jobs	4
MLP	Hidden_layer_sizes	300

**Table 4:** The data test with different deep learning models.

Models	Accuracy (%)	Accuracy Standard Deviation (%)
LR	90.0	4.5
SVM	90.6	5.3
RF	81.9	3.2
KNN	83.0	5.4
MLP	89.8	3.5
GNB	78.0	4.3

**Table 5:** The data test with different deep learning models.

Models	Accuracy (%)	Accuracy Standard Deviation (%)
LR	90.0	4.5
SVM	90.6	5.3
MLP	89.8	3.5
Stacking Model	90.9	4.0
